# Supplementary material for: The structure of a polygamous repressor reveals how phage-inducible chromosomal islands spread in nature
Source: Nat Commun. 2019 Aug 15;10:3676. doi: 10.1038/s41467-019-11504-2 (PMC6695447; doi:10.1038/s41467-019-11504-2)
Supplement: Supplementary file 1 — Supplementary Information [file 41467_2019_11504_MOESM1_ESM.pdf]

Supplementary Information

The structure of a polygamous repressor reveals how phage-inducible chromosomal islands spread in nature.

J. Rafael Ciges-Tomas, Christian Alite, Suzanne Humphrey, J. Donderis, Janine Bowring, Xavier Salvatella, José R Penadés, Alberto Marina.

Supplementary Note 1. Trimeric and dimeric Duts are structurally unrelated proteins.

Trimeric Duts are all-β proteins with three independent active sites formed by the contribution of five conserved motifs (named I-V), with each subunit participating in the formation of each active site present in the trimer[1] (Supplementary Fig. 1). Conversely, dimeric Duts are all-α proteins and their active centers are also generated by five conserved motifs, although the sequences of these motifs are totally different from those on trimeric Duts[2,3] (Supplementary Fig. 1). These differences are reflected in the alternative architectures of the corresponding active centers (Supplementary Fig. 1) and, consequently, different catalytic mechanisms for each family of Duts[2,4,5].

Supplementary Note 2. Changes induced after BovI-Stl$^{N\text{-}ter}$ Dutϕ11 complex formation.

For Dutϕ11, the free (PDB 4GV8)[6] and Stl-bound structures are virtually identical (RMSD of 0.16 Å for the superimposition of 161 residues) (Supplementary Fig. 6a). On the other hand, the BovI-Stl$^{N\text{-}ter}$ shows a small rotational movement (around 18 degrees, calculated with DynDom 3D[7]) between the HTH and middle domains, in which the short α6 acts as a hinge (Supplementary Fig. 6b), supporting some independence between both domains. Individual comparison of each BovI-Stl$^{N\text{-}ter}$ domain in the free and bound structures showed that the HTH domain (RMSD of 1.19 Å for 55 residues) suffers a higher number of local re-organizations than the middle domain (RMSD of 0.58 Å for 60 residues), which mainly affects the DNA recognition helix α3 and the following loop connecting α4 that is partially disordered. As was observed in the free structure, the first nine and the last two residues of the BovI-Stl$^{N\text{-}ter}$ were also disordered, indicating that the complex formation does not stabilize this part of the protein. Similarly, the fifteen C-terminal residues of Dutϕ11, which correspond to the conserved C-terminal P-loop motif V that acquires a stable conformation once the nucleotide binds the enzyme[1,8], were also disordered.

Supplementary Note 3. Stl belongs to HTH XRE-family, permitting modelling of an Stl-DNA complex.

DALI server[9] screening using BovI-Stl HTH domain as a template revealed high structural homology with several members of the HTH XRE-family like proteins (SMART accession number SM00530 or PFAM HTH_3), for example, the 434 Cro repressor family. The superimposition of the

BovI-StI HTH domain with the corresponding HTH domains of these DNA-binding proteins (around 50 to 63 Cα) showed root-mean-square deviations (RMSDs) between 1.3-2.3 Å. In particular, BovI-StI HTH domain superimposed well with the bacterial restriction-modification (R-M) controller proteins (Supplementary Fig. 3a). Superimposition of the HTH domain of BovI-StI$^{N\text{-}ter}$ with the equivalent portion of the controller protein from the Esp1396I R-M system (C.Esp1396I) in complex with DNA (PDB 3CLC)[10] allowed us to generate a model of the StI bound to its target DNA. Since StI and C.Esp1396I present similar DNA binding site architecture (a 4-5 nucleotide "spacer" separating two pseudo-palindromic recognition sites)[11,12], the superimposition of two BovI-StI$^{N\text{-}ter}$ on the equivalent HTH domains of C.Esp1396I was used to generate a biological model of StI-DNA dimer (Fig. 2a and Supplementary Fig. 3b) that revealed residues predicted to be responsible for recognition and interaction with DNA (Fig. 2b).

Supplementary Discussion.

Induction of genes under the repression of several Cro family regulators, such as SinR or bacteriophage 434 CI, involves disruption of the quaternary interactions mediated by the carboxy-terminal domains[13,14]. Prophage induction occurs as a result of the proteolytic cleavage between their N-terminal DNA-binding and C-terminal dimerization domains[13]. The proteolytically separated domains cannot dimerise and no longer bind the operator DNA cooperatively. Although the de-repression of SaPIbov1 is also produced by the disruption of the StI repressor dimer, in this case the mechanism is different since it is mediated by an interaction with the Dut and not by the proteolysis of the repressor. Keeping the StI repressor intact allows the process of de-repression to be transitory; repression could be reverted if the StI-Dut heterodimer was broken, opening the possibility for finely tuned regulation of this process. Our and others' results support this idea, since we have shown that the dUTP substrate acts as an inhibitor of StI binding to both dimeric and trimeric Duts, with the nucleotide being able to act as a switcher of SaPI de-repression[15,16]. In addition, this mechanism of repression requires weak dimers that allow quick conversion to monomers and the consequent exposure of residues involved in anti-repressor recognition. Supporting this, and in difference to the canonical Cro family repressors, the StI construct including the N-terminal and middle domains was monomeric in solution. This indicates that the middle

domain has a weak dimerization capacity that could be attributed to the high hydrophilic character of helix $\alpha 5$. In canonical repressors the helix $\alpha 5$ is the main dimerization element, but in the Stl the presence of the additional C-terminal domain also contributes to dimerization, compensating for the weak capacity of the middle domain. By distributing multiple weak interaction areas along its dimerization surface, Stl has been able to balance its activity as repressor, which requires a stable dimeric organization, with its promiscuity in activation involving the sensing of multiple unrelated protein inducers.

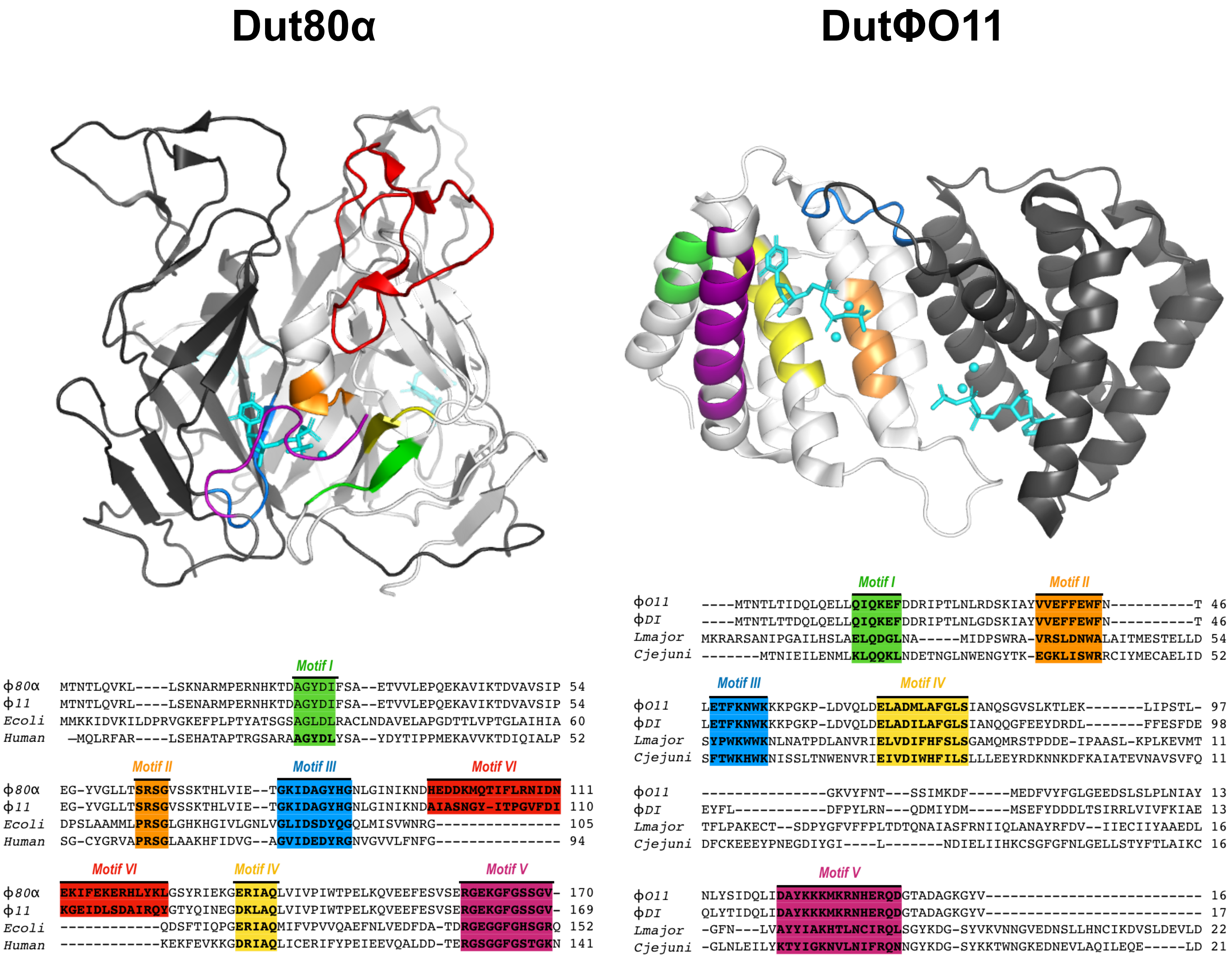

Supplementary Figure 1. Conserved catalytic motifs in trimerics and dimerics Duts. *Top*. The disposition of catalytic motifs of (left) trimeric and (right) dimeric Duts are highlighted on the structures of Dut80α and Dutϕ O11, respectively. dUTP and Mg molecules on the active sites are shown in cyan sticks and balls, respectively. *Bottom*. These motifs are also highlighted with the same colors and named in the alignment of representative Duts from *Staphylococus aureus* phages ϕ80α and ϕ11, *Escherichia coli* and *Homo sapiens* for trimeric Duts and *S. aureus* phages ϕO11 and ϕDI, *Leishmania major* and *Campylobacter jejuni* for dimeric Duts. Note the presence of motif VI (red) characteristic of trimeric Duts encoded by *S. aureus* phages

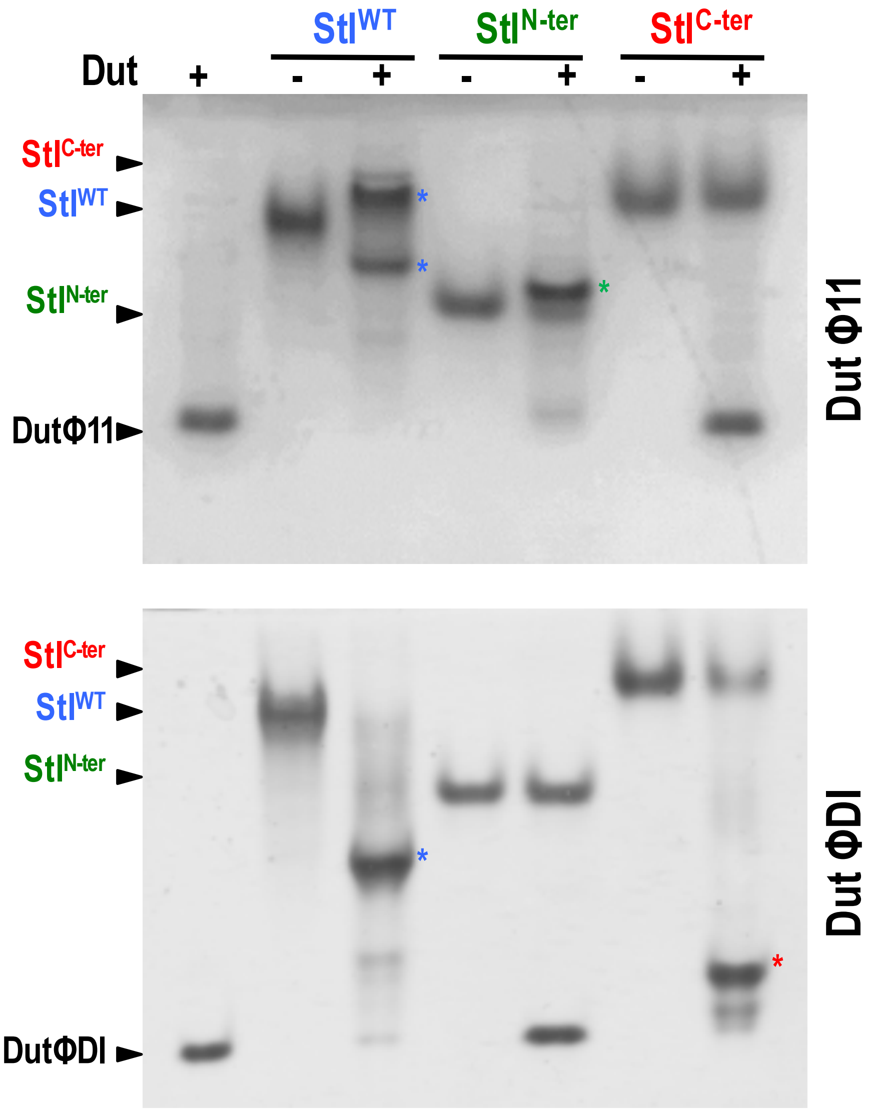

Supplementary Figure 2. Interaction of Trimeric and Dimeric Duts with Stl and its N- and C-terminal portions. Native-PAGE assays were used to test the interaction of the trimeric and dimeric Duts from phages Φ11 and Φ DI, respectively, with the full length Stl (Stl^{WT}), as well as with the Stl N-terminal (residues 1-156, Stl^{N-ter}) and C-terminal (residues 175-267, Stl^{C-ter}). The appearance of a new band (denoted with an asterisk coloured as the corresponding Stl) concomitant with the disappearance of the bands corresponding to the individual proteins confirms the formation of the Stl-Dut complex. For trimeric DutΦ11 the N-terminal portion is sufficient to form the complex, while the complex formation for dimeric DutΦDI requires the C-terminal portion. Source data are provided as a Source Data file.

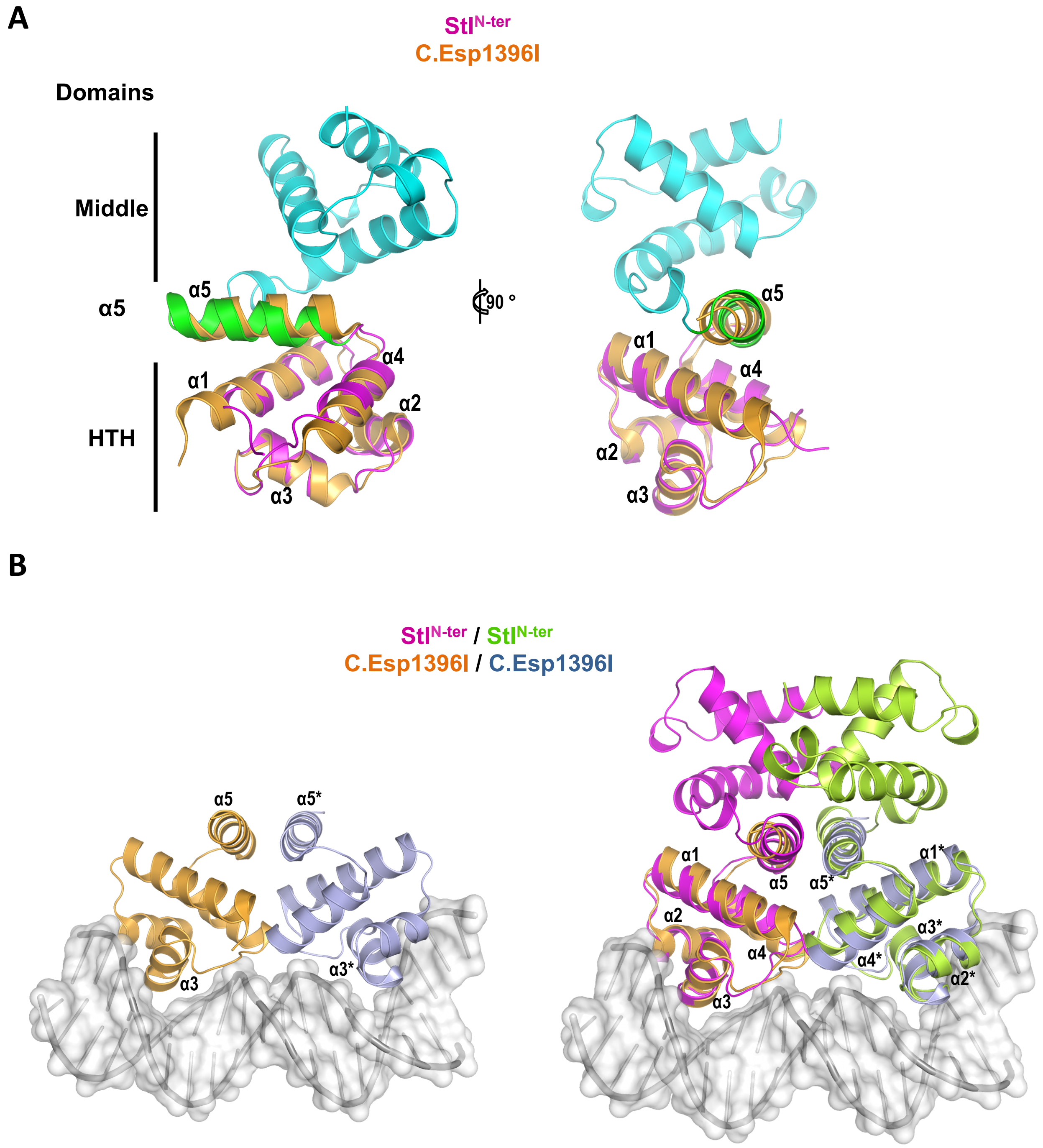

Supplementary Figure 3. Comparison of the structure of Stl with the controller C-Esp1396I. (A) The superimposition of Stl$^{N\text{-}ter}$ and C.Esp1396I (PDB 3CLC[10]), the controller protein of the restriction-modification system Esp1396I, shows that the structural identity between both proteins not only includes the HTH DNA-binding domains (purple and orange for Stl and C.Esp1396I, respectively) but also the following helix α5 (green in Stl), which is used in Stl to connect the HTH with the middle domain (cyan). Both proteins are represented in ribbon and two orthogonal views are shown. The α helices composing the HTH domain are labelled. (B) *left,* the structure of the dimeric C.Esp1396I (protomers in orange and blue) bound to DNA ((white) PDB 3CLC) was used to generate a model of Stl$^{N\text{-}ter}$-DNA by superimposing two monomers (magenta and green) onto the equivalent regions of C.Esp1396I (*right*). The common structural elements are numbered and labelled. The asterisks denote the elements of the second protomer.

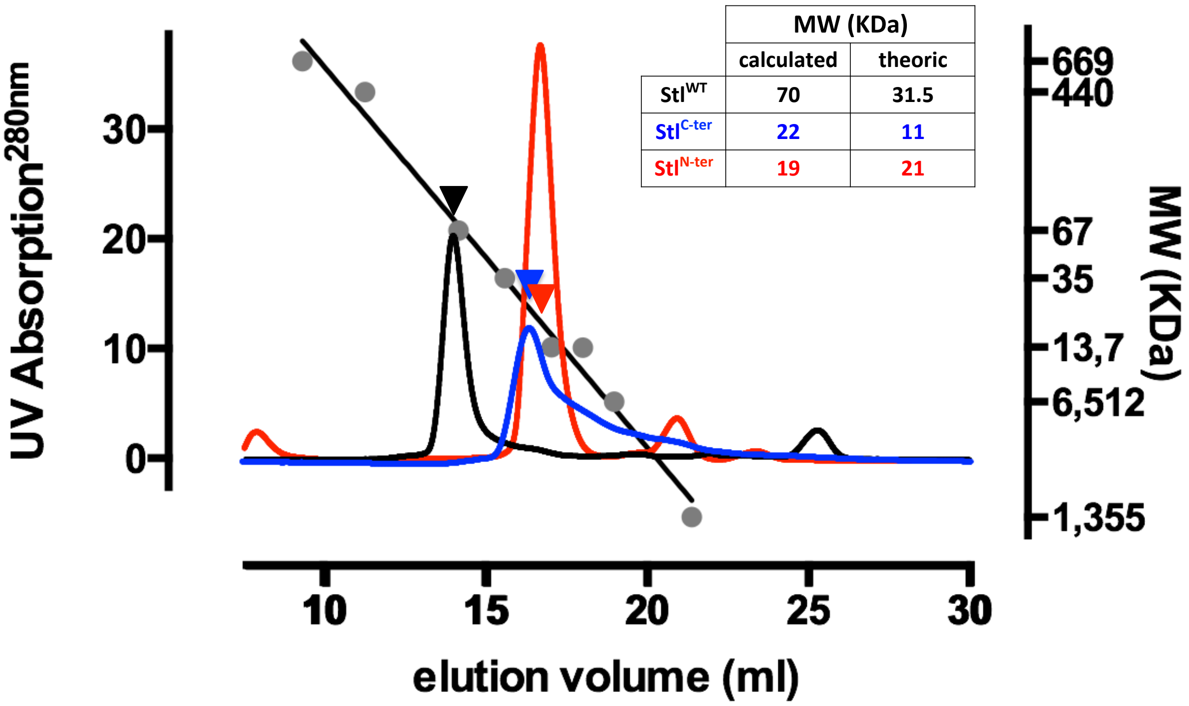

Supplementary Figure 4. Size exclusion chromatography of Stl^{WT}, Stl$^{N\text{-}ter}$ and Stl$^{C\text{-}ter}$. Elution profiles of the proteins were monitored as UV absorption (black, red and blue curves) and plotted semilogarithmically (black, blue and red triangles) together with the elution positions of molecular mass standards (gray circles). The molecular weight was deduced from the elution volumes (inset) and supports a dimeric organization for Stl^{WT} and Stl$^{C\text{-}ter}$ and monomeric for Stl$^{N\text{-}ter}$. Source data are provided as a Source Data file.

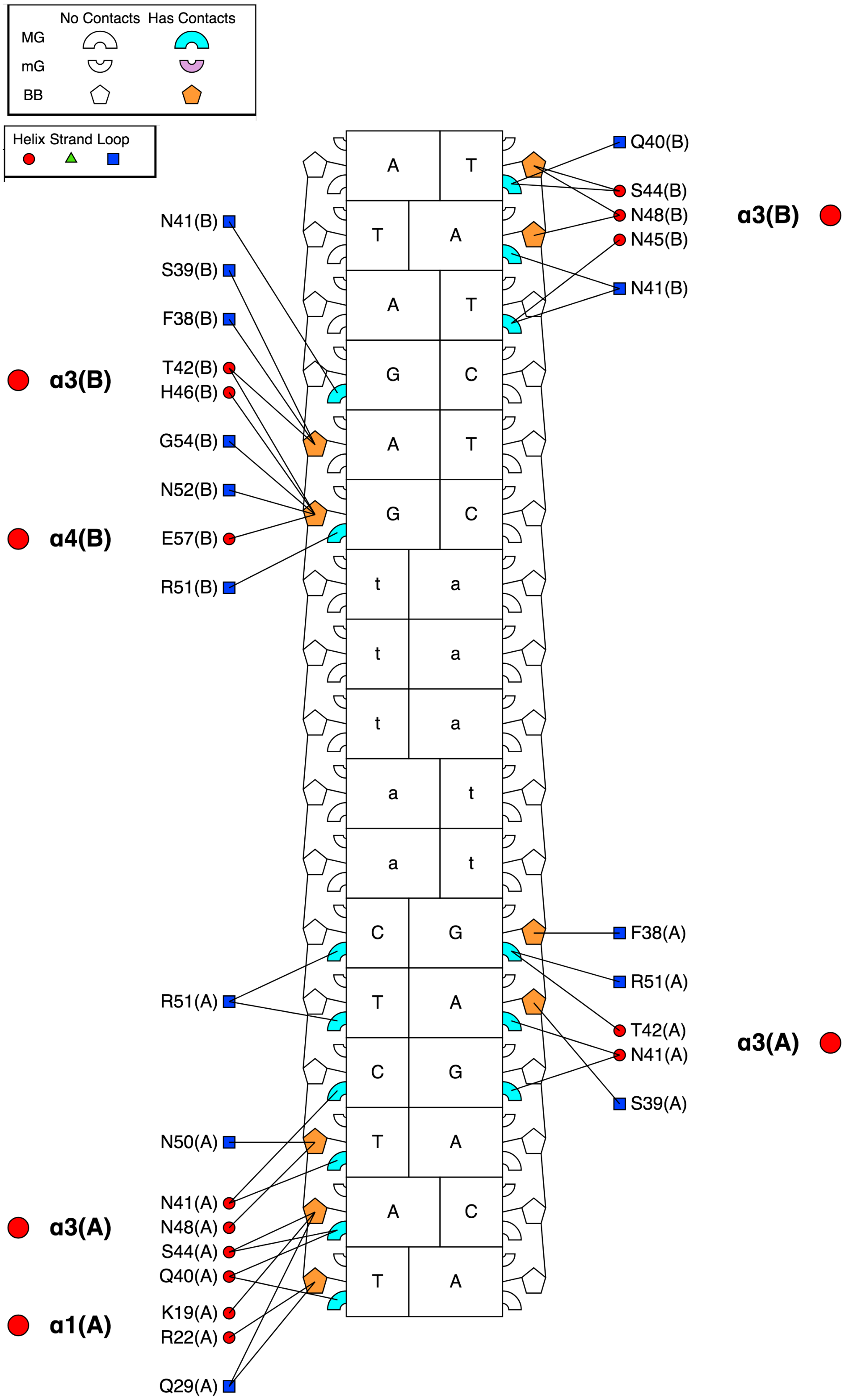

Supplementary Figure 5. **Nucleotide-residue contact map.** Schematic representation from DNAproDB[17] showing the individual protein-nucleotide interactions observed in the Stl-DNA model. The Stl palindromic DNA recognition sequence (TATCTC) is highlighted in capital letters. Specific read-out through major groove (MG) interactions are highlighted in large markers filled-in cyan while nonspecific interactions with the DNA backbone (BB) are highlighted with pentagons filled-in orange. Protein helices and loop residues are depicted in red circles and blue squares, respectively.

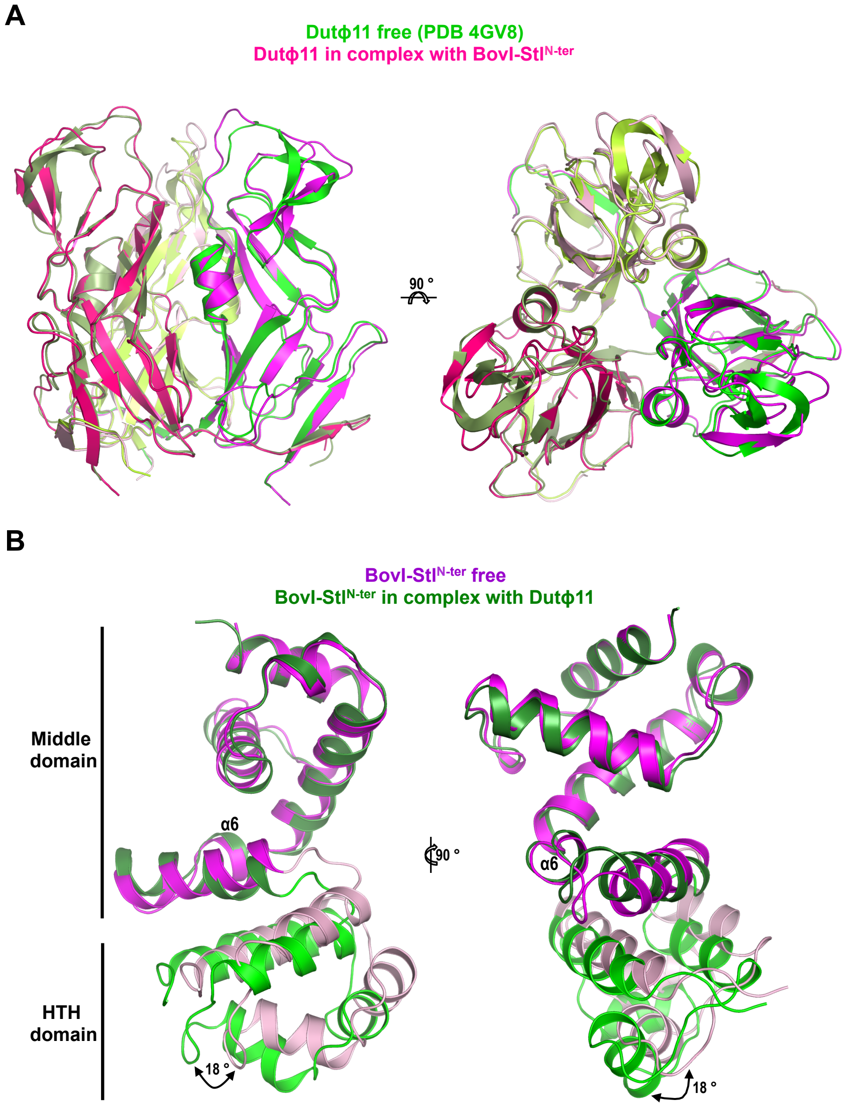

Supplementary Figure 6. Conformational changes in BovI-Stl$^{N\text{-}ter}$ and Dutϕ11 induced by complex formation. (A) The crystal structures of the trimer of Dutϕ11 both in complex with BovI-Stl$^{N\text{-}ter}$ and in free form were overlaid, and two orthogonal views in cartoon representation are displayed, with the protomers of the free form in different tones of green and the protomers of the Dut in complex with BovI-Stl$^{N\text{-}ter}$ in different tones of magenta. (B) The structures of BovI-Stl$^{N\text{-}ter}$ obtained in its free form (green) or in complex with Dutϕ11 (magenta) were overlaid and two orthogonal views are shown in cartoon representation. The HTH domain is coloured in a lighter tone.

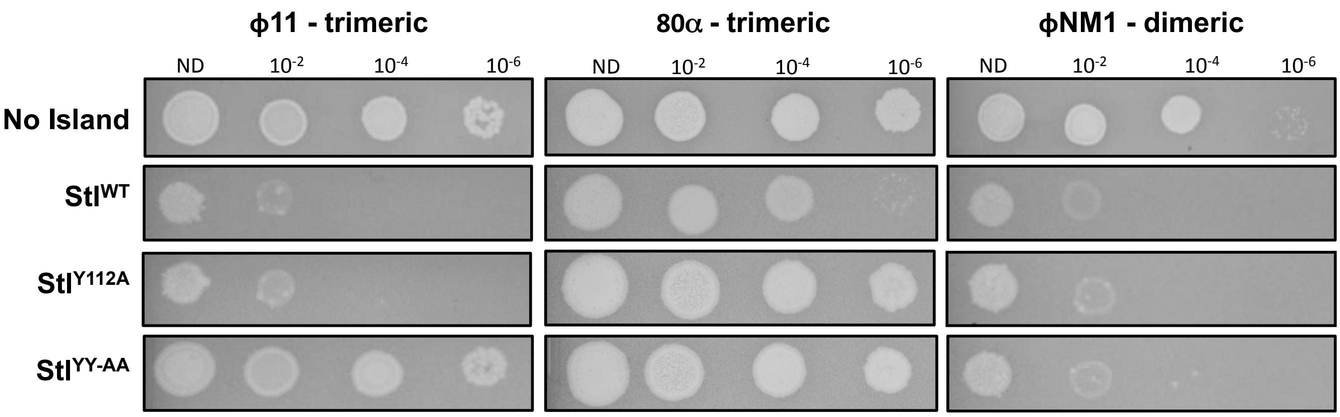

Supplementary Figure 7. Phage spotting assay using the trimeric Dut encoding phages ϕ11 and 80α, and dimeric Dut encoding ϕNM1 on bacterial lawns of RN4220 strains either without any SaPI, with the SaPIbov1 Stl^{WT}, or with SaPIbov1 mutants Stl^{Y112A} or $Stl^{YY\text{-}AA}$. Serially diluted (10-fold) lysates were spotted on the lawns of indicated *S. aureus*. The phage titre (PFU/ml) used for each spot is indicated. Source data are provided as a Source Data file.

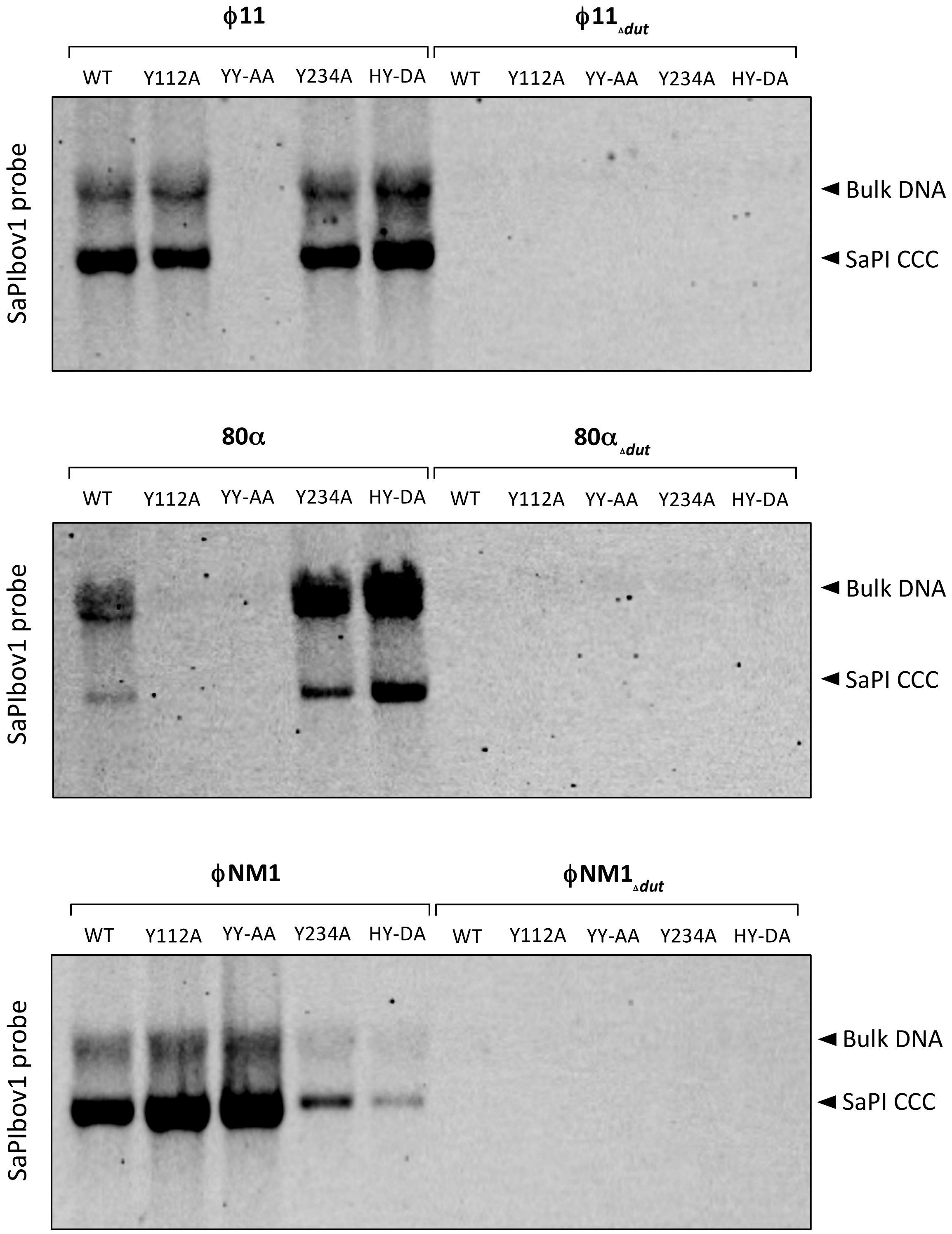

Supplementary Figure 8. *In vivo* effects of Stl mutations on SaPIbov1 excision and replication following helper phage activation. Strains JP6774, JP17043, JP17706, JP18043, and JP17679 containing SaPIbov1^{WT}, Stl^{Y234A}, Stl^{Y112A}, Stl^{YY-AA} and Stl^{HY-DA} respectively were lysogenised with either phage ϕ11 (trimeric Dut), phage 80α (trimeric Dut), or ϕNM1 (dimeric Dut), or with their *Δdut* derivatives. Samples were isolated 90 mins post-induction with 2 μg/ml MC and Southern blots were performed using a SaPIbov1 integrase probe. The upper band is 'bulk' DNA, including chromosomal, phage, and replicating SaPI. CCC indicates covalently closed circular SaPI DNA. Source data are provided as a Source Data file.

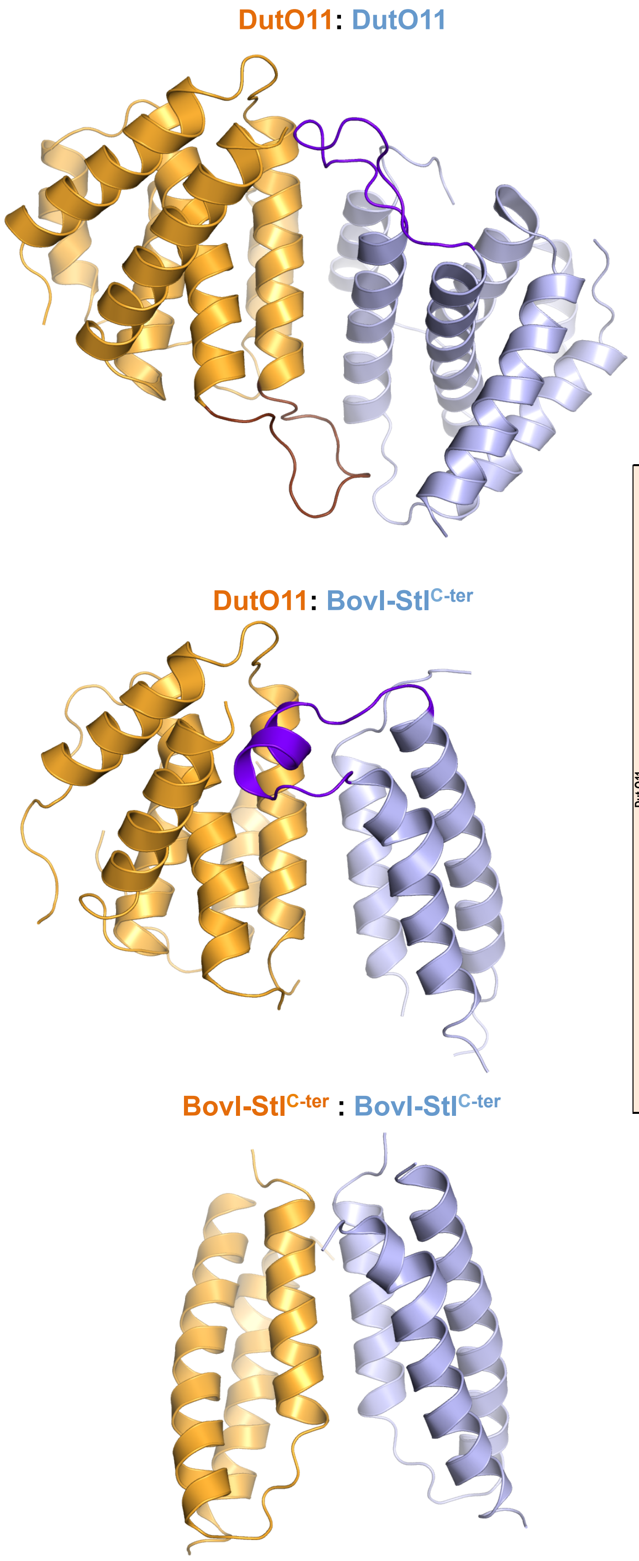

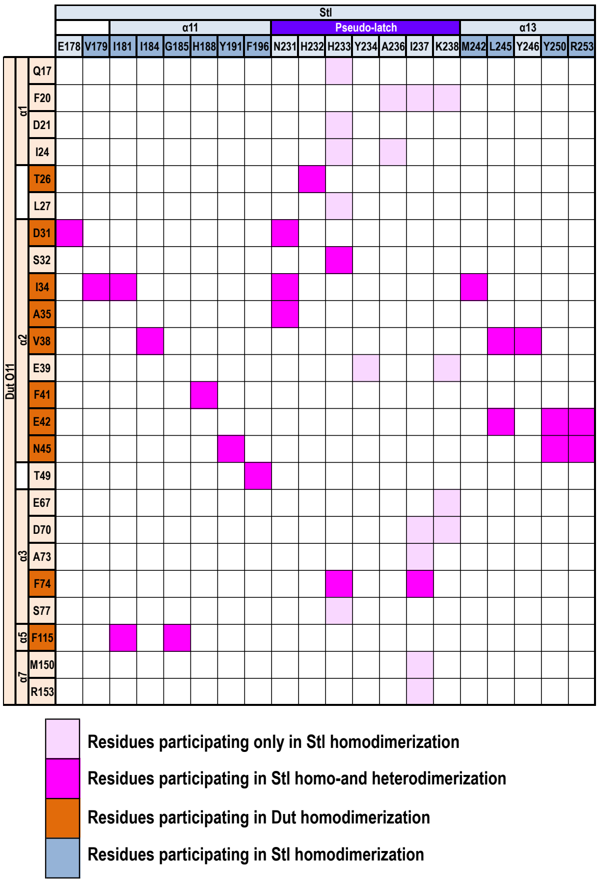

Supplementary Figure 9. BovI-Stl$^{C\text{-}ter}$ mimics the partner protomer in dimeric Duts. *Left*, Cartoon representation of the structures of DutϕO11 (PDB 5MIL; [18]) (top) and BovI-Stl$^{C\text{-}ter}$ (bottom) homodimers and a BovI-Stl$^{C\text{-}ter}$-DutϕO11 heterodimer (middle) in identical orientation by overlaying one homodimer protomer on the corresponding component on the heterodimer structure. Notice that to form the BovI-Stl$^{C\text{-}ter}$- DutϕO11 heterodimer the BovI-Stl$^{C\text{-}ter}$ protomer occupies an identical position to the second protomer of the DutϕO11 homodimer, mimicking the interaction of this protomer in the complex formation. *Right*, Contact map of BovI-Stl$^{C\text{-}ter}$ and DutϕO11. The contacts between DutϕO11 (Y axis) and BovI-Stl$^{C\text{-}ter}$ (X axis) residues are denoted by coloured squares (contacts calculated with Contact Software). Magenta squares indicate contacts between residues that also mediate interactions in the homodimerization of both proteins. Pink squares denote contacts between residues that do not participate in homodimerization interactions of any of the proteins. Brown and blue squares are for contact of residues that also participate in the homodimerization of DutϕO11 and BovI-Stl$^{C\text{-}ter}$, respectively. The structural elements where the contacting residues are localized in each protein are indicated in the axis of the map.

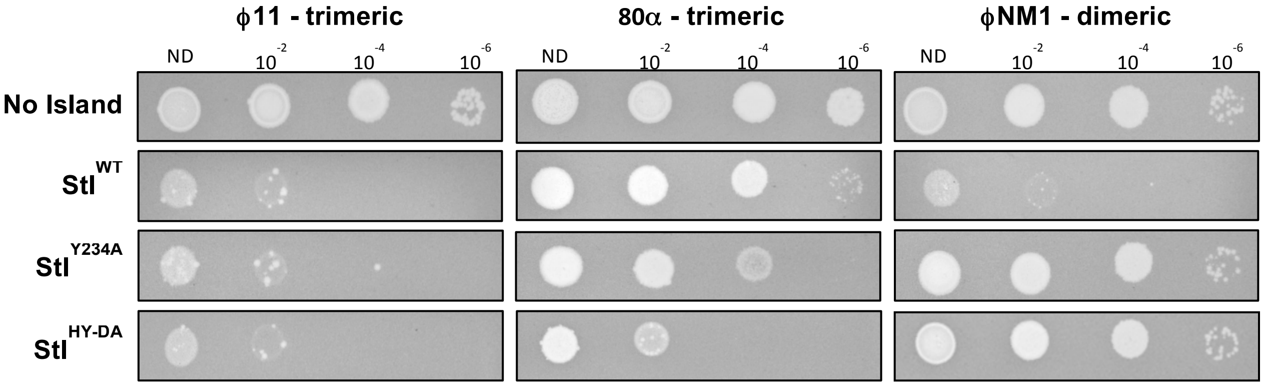

Supplementary Figure 10. Phage spotting assay using the trimeric Dut encoding phages ϕ11 and 80α, and dimeric Dut encoding ϕNM1 on bacterial lawns of RN4220 strains either without any SaPI, with the SaPIbov1 Stl^{WT}, or with SaPIbov1 mutants Stl^{Y234A} or $Stl^{HY\text{-}DA}$. Serially diluted (10-fold) lysates were spotted on the lawns of indicated *S. aureus*. The phage titre (PFU/ml) used for each spot is indicated on the plate. Source data are provided as a Source Data file.

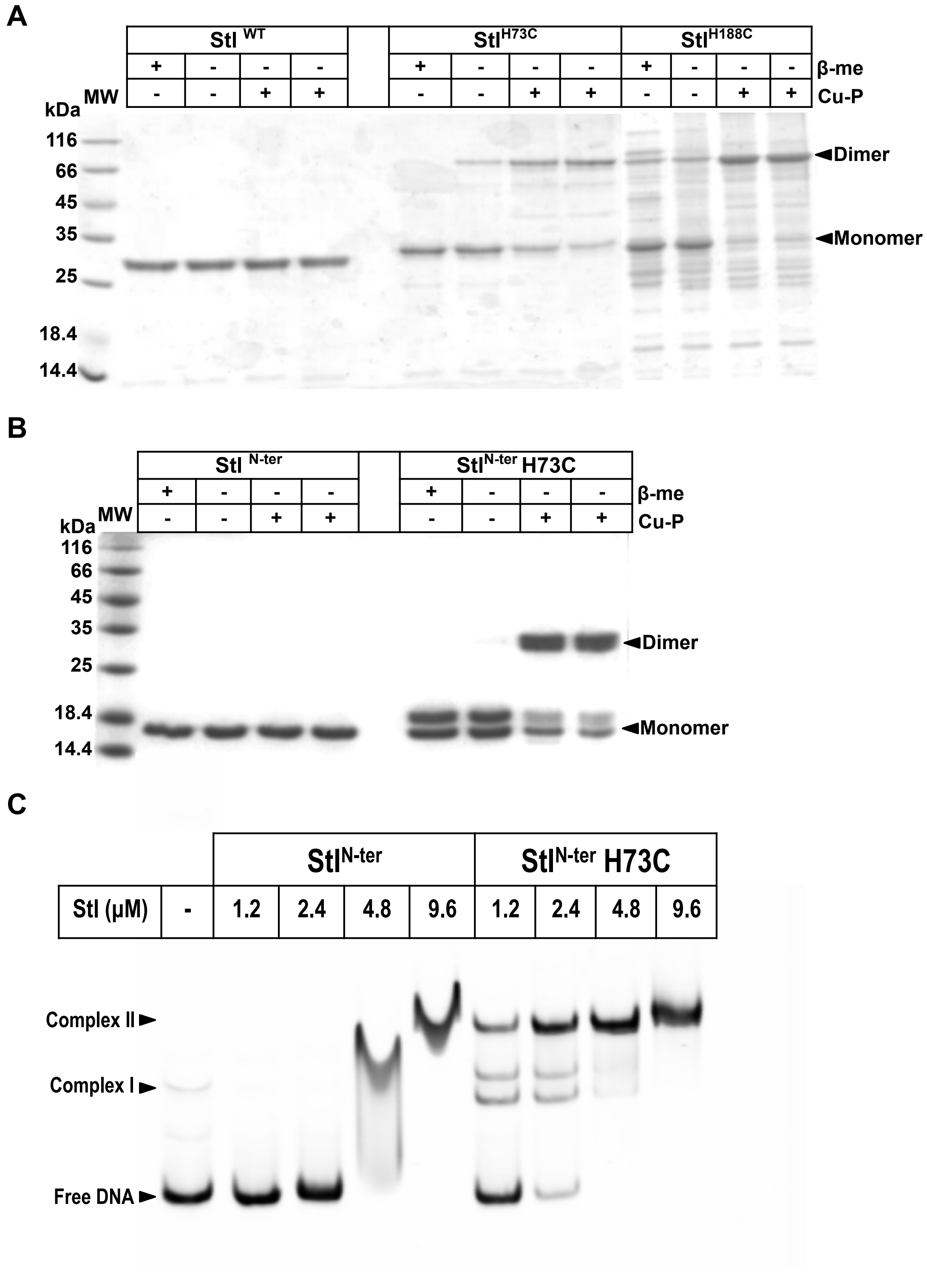

Supplementary Figure 11. Crosslinked dimers of Stl. (A) BovI-Stl His 73 or 188 were mutated to Cys to facilitate the production of disulphide crosslinked dimers. The formation of crosslinked dimers was induced by the addition of the oxidizing reagent copper-phenantroline (Cu-P; 0.5 and 1 mM) and the production of disulphide bound dimers was evaluated by SDS-PAGE in the presence or absence of the reducing agent β-mercaptoethanol (β-me). (B) The formation of dimers induced by the mutation of His73 to Cys was also analysed in the context BovI-Stl$^{N\text{-}ter}$ using an identical approach. (C) EMSA assays with Stl$^{N\text{-}ter}$ and the H73C mutant of this construct show that the forced dimeric H73C mutant produces a shifted band corresponding to the DNA-Stl complex at lower concentrations of Stl protein, supporting a higher affinity for DNA of the Stl H73C mutant. Source data are provided as a Source Data file.

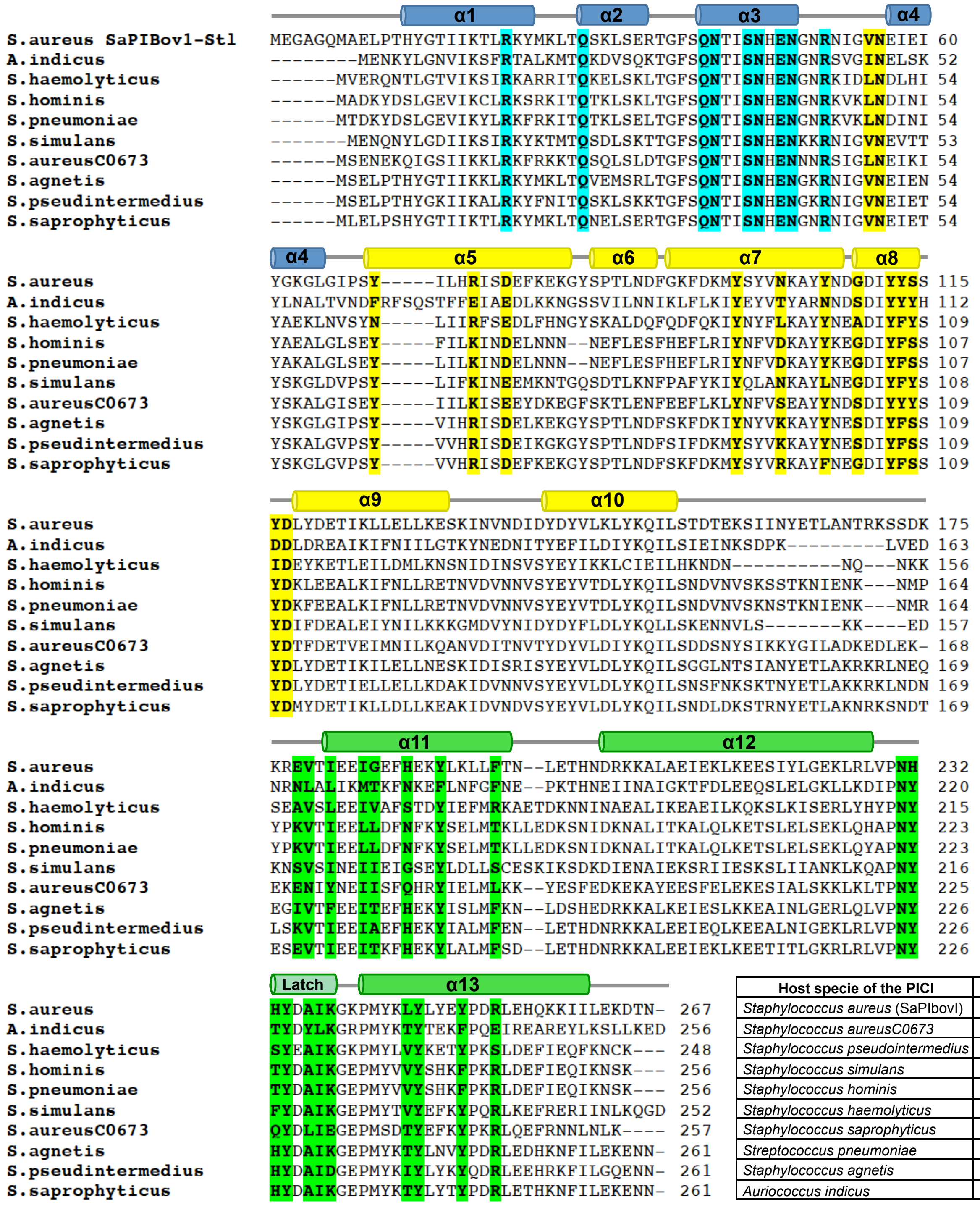

Host specie of the PICI	Stl-like acession
Staphylococcus aureus (SaPIbovI)	AAG29617.1
Staphylococcus aureusC0673	EZX20759.1
Staphylococcus pseudointermedius	PKW51886.1
Staphylococcus simulans	AMG96720.2
Staphylococcus hominis	WP_049379208
Staphylococcus haemolyticus	WP_053031052.1
Staphylococcus saprophyticus	OEK45527.1
Streptococcus pneumoniae	CVY54148.1
Staphylococcus agnetis	PNY87679.1
Auriococcus indicus	AQL56990.1

Supplementary Figure 12. Clustal alignment of SaPIbov1 Stl with homologous Stl repressors from PICIs of different species. *Auriococcus indicus* Stl sequence is included as external genus within the *Staphylococcaceae* family and *Streptococcus pneumoniae* representing a more distanced species from the *Lactobacillales* order. The residues participating in DNA recognition (highlighted with blue background) are completely conserved, whereas the residues involved in the recognition of trimeric (yellow) or dimeric (green) Duts are partially conserved among Stl repressors. Secondary structural elements are represented above the SaPIbov1 Stl sequence and coloured in blue, yellow and green for the HTH DNA binding, middle and C-terminal domains, respectively. Data base accession code for the corresponding Stl sequences are indicated in the inset table.

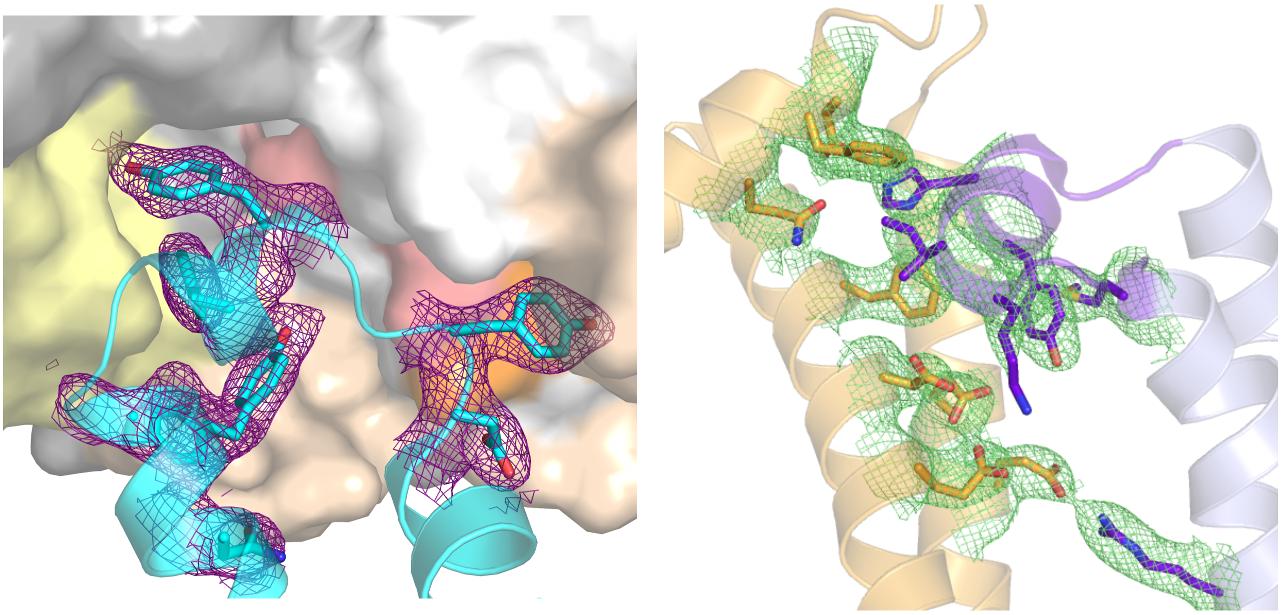

Supplementary Figure 13. Omit maps for interacting residues in the complexes of Stl with trimeric and dimeric Duts. The electron-density omit map (contour level: 1σ) are shown in (*left)* magenta and (*right)* green around (2 Å) the interacting residues of the complexes of Stl with trimeric and dimeric Duts showed in Figures 3c and 5c, respectively. Structures are represented as in the corresponding Figures.

Supplementary Table 1. Intersubunit interactions for Stl$^{C\text{-}ter}$ dimer

Stl$^{C\text{-}ter}$ Subunit A			Stl$^{C\text{-}ter}$ Subunit B			
**Structural element**	**Residue**	**Atom type**	**Structural element**	**Residue**	**Atom type**	**Distance (Å)**
α11	181 (ILE)	CG2	α11	181 (ILE)	CG2	3.53
		O	α13	242 (MSE)	CE	3.47
		CB	α11	184 (ILE)	CD1	3.45
		CG2			CD1	3.88
		CD1	α12	226 (LEU)	CD2	3.81
	184 (ILE)	CG2	α11	184 (ILE)	CG2	3.55
		CD1		181 (ILE)	CB	3.45
		CD1			CG2	3.88
	185 (GLY)	CA	α13	242 (MSE)	CE	3.55
	188 (HIS)	CB	α11	188 (HIS)	CG	3.50
		CB			ND1	3.51
		CB			CD2	3.69
		CB			CE1	3.69
		CB			NE2	3.79
		CG			CG	3.43
		CG			CD2	3.76
		CD2			CD2	3.79
	189 (GLU)	OE1	α13	250 (TYR)	OH	2.19
		OE1		253 (ARG)	NH2	3.73
		OE2			NH2	2.95
	191 (TYR)	OH	α11	196 (PHE)	CZ	3.73
	192 (LEU)	CD1	α13	250 (TYR)	CE1	3.51
		CD1			CZ	3.80
		CD2	α11	195 (LEU)	CD1	3.71
	193 (LYS)	NZ	α13	253 (ARG)	NH2	3.87
	195 (LEU)	CD1	α11	192 (LEU)	CD2	3.71
	196 (PHE)	CD2	α13	253 (ARG)	NH1	3.37
		CE2			CD	3.27
		CE2			NH1	3.29
		CZ	α11	191 (TYR)	OH	3.73
α12	226 (LEU)	CD2		181 (ILE)	CD1	3.81
α13	242 (MSE)	CE		185 (GLY)	CA	3.55
	245 (LEU)	CD2		189 (GLU)	OE1	3.64
	250 (TYR)	OH			OE1	2.19
		CE1		192 (LEU)	CD1	3.51
		CZ			CD1	3.80
	253 (ARG)	CD		196 (PHE)	CE2	3.27
		NH1			CD2	3.37
		NH1			CE2	3.29
		CZ		189 (GLU)	OE2	3.83
		NH2			OE1	3.73
		NH2			OE2	2.95
		NH2		193 (LYS)	NZ	3.87

Supplementary Table 2. Molecular weight and thermostability of BovI-Stl WT and mutants

Protein	Molecular Weight (kDa)[a]	Tm (˚C)[b]
Stl^{WT}	61,4 ± 1,23	54,7 ± 0,18
Stl^{R22A}	66,3 ± 2,12	55,6 ± 0,11
Stl^{Q29A}	65,8 ± 1,58	52,9 ± 0,17
Stl^{S44A}	64,1 ± 3,53	55,1 ± 0,10
Stl^{N45A}	63,4 ± 3,74	55,4 ± 0,08
Stl^{E47A}	66,7 ± 1,93	54,6 ± 0,12
Stl^{N48A}	70,2 ± 1,26	54,1 ± 0,19
Stl^{R51A}	65,1 ± 1,37	54,8 ± 0,16
Stl^{R74A}	69,0 ± 2,21	50,8 ± 0,22
Stl^{Y112A}	68,6 ± 1,99	54,8 ± 0,18
Stl^{Y113A}	68,8 ± 1,38	55,5 ± 0,09
Stl^{YY-AA}	69,4 ± 2,91	58,1 ± 0,24
Stl^{Y116A}	72,7 ± 4,29	55,9 ± 0,16

[a]Molecular weight of each protein was calculated by SEC-MALS

[b]Thermostability of each protein was calculate by Thermofluor

Supplementary Table 3. Intermolecular interactions for Stl$^{N\text{-}ter}$ – Dut ϕ11 complex

Stl$^{N\text{-}ter}$			Dut ϕ11			
**Structural element**	**Residue**	**Atom type**	**Structural element**	**Residue**	**Atom type**	**Distance (Å)**
α4	55 (VAL)	CB	β11	149 (GLU)	CD	3.89
	56 (ASN)	OD1			N	2.85
		ND2			O	2.85
α5	70 (TYR)	OH	Lβ1β2	20 (ASN)	ND2	3.72
		CB		18 (GLU)	CD	3.46
					CG	3.78
	74 (ARG)	NH1			OE1	2.41
					OE2	3.39
					CD	3.27
	77 (ASP)	OD1		15 (ARG)	NH1	3.69
		OD2			NH2	3.04
α6	98 (TYR)	OH		20 (ASN)	OD1	3.11
	102 (ASN)	OD1				3.49
	106 (TYR)	CE2		21 (HIS)	CE1	3.66
					ND1	3.72
		CZ				3.70
		OH				3.19
				24 (ASP)	OD1	2.78
					OD2	3.14
		O	Lβ7β8	85 (HIS)	N	3.08
α7	109 (GLY)	N		84 (TYR)	OH	3.62
	112 (TYR)	OH	β7	79 (LYS)	O	3.50
			Lβ7β8	81 (ASP)	N	3.11
					OD2	2.81
		CB		84 (TYR)	CE1	3.37
		CG				3.27
					CD1	3.36
		CD1				3.51
		CD2				3.47
		CE2				3.57
					CG	3.73
		CZ				3.71
					CB	3.78
	113 (TYR)	O	α1	65 (SER)	O	3.49
				66 (GLY)	N	3.55
				69 (SER)	OG	3.05
		CE2	β8	89 (GLY)	N	3.61
		CZ				3.39
		OH				2.95
	114 (SER)	CB	α1	70 (TYR)	CD	3.88
Lα7α8	116 (TYR)	O		64 (ARG)	NH1	3.35
					NH2	2.65
		C				3.54
		CD1				3.40
		CE1	Lβ8β9	110 (ILE)	CG2	3.59
		CE2		111 (LYS)	CE	3.83
		CZ	Lβ9β10	133 (LYS)	N	3.81
					CB	3.96
	117 (ASP)	OD1			NZ	2.76
α9	152 (LEU)	O	α1	70 (TYR)	NZ	3.03

Supplementary Table 4. Interactions of dUTP-Mg with residues of trimeric DutΦ11 (PDB 4GV8) and dimeric DutΦO11 (PDB 5MIL) and interactions of these residues with Stl in the corresponding complexes.

		DutΦ11-$Stl^{N\text{-}ter}$ complex
DutΦ11-dUTP complex PDB 4GV8		
**dUTP**	**DutΦ11**	**$Stl^{N\text{-}ter}$**
**Structural element**	**Residue**	**Residue**
Pγ	20 (ASN)	70 (TYR)
	64 (ARG)	116 (TYR)
Pβ		
Pα	65 (SER)	113 (TYR)
Deoxyribose		
Uracil		
Pβ	66 (GLY)	
Uracil	78 (GLY)	-
Deoxyribose	79 (LYS)	112 (TYR)
Uracil	80 (ILE)	
Deoxyribose		
	81 (ASP)	
Pα		
Deoxyribose	84 (TYR)	109 (GLY)
Uracil		112 (TYR)
	89 (GLY)	Y113 (TYR)
Pα	136 (GLN)	-

		DutΦ11-$Stl^{C\text{-}ter}$ complex
DutΦO11-dUTP complex PDB 5MIL		
**dUTP**	**DutO11**	**$Stl^{C\text{-}ter}$**
**Structural element**	**Residue**	**Residue**
Deoxyribose	17 (GLN)	233 (HIS)
	20 (PHE)	237 (ILE)
		236 (ALA)
	21 (ASP)	233 (HIS)
	27 (LEU)	
Mg ion	39 (GLU)	238 (LYS)
		234 (TYR)
Pγ	42 (GLU)	245 (LEU)
Mg ion		
Pγ		250 (TYR)
Mg ion		
Pβ		253 (ARG)
Pγ		
Mg ion		
Pγ	45 (ASN)	191 (TYR)
		250 (TYR)
		253 (ARG)
Mg Ion	67 (GLU)	238 (LYS)
Deoxyribose	70 (ASP)	237 (ILE)
Mg ion		
Deoxyribose		238 (LYS)
Pβ		
Mg ion		
Deoxyribose	73 (ALA)	237 (ILE)
Deoxyribose	74 (PHE)	237 (ILE)
Uracil		
Uracil		233 (HIS)
Deoxyribose		
Deoxyribose	153 (ASN)	237 (ILE)

Supplementary Table 5. Effect of Stl mutations on SaPIbov1 titre.

		SaPI titre[a]				
**Dut family**	**Helper phage**	**Stl^{WT}**	**Stl^{Y112A}**	**$Stl^{YY\text{-}AA}$**	**Stl^{Y234A}**	**$Stl^{HY\text{-}DA}$**
Trimeric	ϕ11	10.06	9.81	2.38****	9.97	10.11
	$\phi 11_{\Delta dut}$	2.35****	2.37****	2.49****	2.54****	2.82****
Trimeric	80α	8.05	1.63****	1.71****	8.60	9.02*
	$80\alpha_{\Delta dut}$	1.87****	1.89****	1.87****	1.90****	2.01****
Dimeric	ϕNM1	9.40	9.90	9.76	7.94***	7.61****
	$\phi NM1_{\Delta dut}$	2.91****	2.85****	2.82****	2.85****	2.97****

[a]Numbers refer to the log_{10} number of transductants in RN4220 recipient per 10^9 phage particles. The means of results from three independent experiments are presented. Variation was within ±5% in all cases.

A 2-way ANOVA with Tukey's multiple comparisons test was performed to compare mean differences between the $phage_{dut+}$ Stl^{WT} control and the mutants for each phage and Stl type. Significant adjusted *p* values were as follows: ϕ11 $Stl^{YY\text{-}AA}$ = <0.0001****, $\phi 11_{\Delta dut}$ Stl^{WT} = <0.0001****, $\phi 11_{\Delta dut}$ Stl^{Y112A} = <0.0001****, $\phi 11_{\Delta dut}$ $Stl^{YY\text{-}AA}$ = <0.0001****, $\phi 11_{\Delta dut}$ Stl^{Y234A} = <0.0001****, $\phi 11_{\Delta dut}$ $Stl^{HY\text{-}DA}$ = <0.0001****; 80α Stl^{Y112A} = <0.0001****, 80α $Stl^{YY\text{-}AA}$ = <0.001****, 80α $Stl^{HY\text{-}DA}$ = 0.024, $80\alpha_{\Delta dut}$ Stl^{WT} = <0.0001****, $80\alpha_{\Delta dut}$ Stl^{Y112A} = <0.0001****, $80\alpha_{\Delta dut}$ $Stl^{YY\text{-}AA}$ = <0.0001****, $80\alpha_{\Delta dut}$ Stl^{Y234A} = <0.0001****, $80\alpha_{\Delta dut}$ $Stl^{HY\text{-}DA}$ = <0.0001; ϕNM1 Stl^{Y234A} = 0.0002***, ϕNM1 $Stl^{HY\text{-}DA}$ = <0.0001****; $\phi NM1_{\Delta dut}$ Stl^{WT} = <0.0001****, $\phi NM1_{\Delta dut}$ Stl^{Y112A} = <0.0001****, $\phi NM1_{\Delta dut}$ $Stl^{YY\text{-}AA}$ = <0.0001****, $\phi NM1_{\Delta dut}$ Stl^{Y234A} = <0.0001****, $\phi NM1_{\Delta dut}$ $Stl^{HY\text{-}DA}$ = <0.0001****, all other values were not significant.

Supplementary Table 6. Intermolecular interactions for $Stl^{C\text{-}ter}$ – Dut ΦO11

$Stl^{C\text{-}ter}$			Dut ϕO11			
**Structural element**	**Residue**	**Atom Type**	**Structural element**	**Residue**	**Atom Type**	**Distance**
α11	178 (GLU)	OE2	α2	31 (ASP)	OD1	3.49
	179 (VAL)	O		34 (ILE)	CD1	3.80
	181 (ILE)	CG1			CG1	3.59
		CG2	α5	115 (PHE)	CE1	3.57
		O			CZ	3.68
	184 (ILE)	CG2	α2	38 (VAL)	CG2	3.88
	185 (GLY)	CA	α5	115 (PHE)	CZ	3.85
	188 (His)	CG	α2	41 (PHE)	CB	3.87
		ND1			CB	3.59
		CB			CG	3.52
		CG			CG	3.81
		CB			CD1	3.81
		CB			CD2	3.59
		CG			CD2	3.73
	191 (TYR)	CE2		45 (ASN)	OD1	3.14
		OH			OD1	3.83
	196 (PHE)	CE1		49 (THR)	C	3.49
		CZ			C	3.84
		CE1			O	3.61
latch	231 (ASN)	CG		31 (ASP)	CB	3.57
		OD1			CB	3.34
		ND2			CB	3.50
		ND2			CA	3.55
		ND2			C	3.59
		CG			O	3.73
		OD1			O	3.69
		ND2			O	2.93
		ND2			OD1	3.77
		ND2		34 (ILE)	CB	3.79
		ND2		35 (ALA)	N	3.76
	232 (HIS)	ND1		26 (THR)	CB	3.56
		CB			OG1	3.25
		CG			OG1	3.05
		ND1			OG1	2.57
		CE1			OG1	3.43
		ND1			CG2	3.74
		CE1			CG2	3.82
		CG		27 (LEU)	CD1	3.67
		CD2			CD1	3.59
		NE2			CD1	3.87
		ND1		32 (SER)	OG	3.37
	233 (HIS)	CE1	α1	17 (GLN)	NE2	3.58
		NE2			NE2	3.43
		NE2		21 (ASP)	CG	3.34
		NE2			OD1	3.05
		CE1			OD2	3.44
		NE2			OD2	3.16
		CD2		24 (ILE)	CD1	3.43
		ND1	α3	74 (PHE)	CD1	3.35
		CE1			CD1	3.46
		O			CE1	3.75
		ND1			CE1	3.38
		CE1			CE1	3.68
		CE1		77 (SER)	CB	3.70
		CE1			OG	3.55
	234 (TYR)	CE2	α2	39 (GLU)	OE2	3.45
		OH			OE2	3.71

	236 (ALA)	O	α1	20 (PHE)	CE2	3.74
	236 (ALA)	CB	α1	20 (PHE)	CE2	3.85
	236 (ALA)	O	α1	20 (PHE)	CZ	3.45
	236 (ALA)	CB	α1	24 (ILE)	CD1	3.63
	237 (ILE)	CD1	α1	20 (PHE)	CD2	3.62
	237 (ILE)	CD1	α1	20 (PHE)	CE2	3.59
	237 (ILE)	O	α3	70 (ASP)	OD1	3.78
	237 (ILE)	CG2	α3	70 (ASP)	OD1	3.86
	237 (ILE)	CG2	α3	73 (ALA)	CB	3.83
	237 (ILE)	CB	α3	74 (PHE)	CZ	3.73
	237 (ILE)	CB	α3	74 (PHE)	CE1	3.80
	237 (ILE)	CG1	α3	74 (PHE)	CE1	3.48
	237 (ILE)	CD1	α7	150 (MET)	CE	3.84
	237 (ILE)	CG	α7	153 (ASN)	O	3.82
	237 (ILE)	OD1	α7	153 (ASN)	O	3.76
	237 (ILE)	ND2	α7	153 (ASN)	O	3.31
	238 (LYS)	CE	α2	39 (GLU)	CD	3.82
	238 (LYS)	CE	α2	39 (GLU)	OE1	3.87
	238 (LYS)	CE	α2	39 (GLU)	OE2	3.20
	238 (LYS)	NZ	α3	67 (GLU)	CD	3.72
	238 (LYS)	CE	α3	67 (GLU)	OE1	3.73
	238 (LYS)	NZ	α3	67 (GLU)	OE1	3.30
	238 (LYS)	NZ	α3	67 (GLU)	OE2	3.40
	238 (LYS)	CD	α3	70 (ASP)	OD2	3.75
	238 (LYS)	CE	α3	70 (ASP)	OD2	3.30
α13	242 (MSE)	CE	α2	34 (ILE)	CG2	3.68
α13	245 (LEU)	CD2	α2	38 (VAL)	CG1	3.70
α13	245 (LEU)	CD2	α2	42 (GLU)	OE2	3.73
α13	245 (LEU)	CD1	α2	42 (GLU)	OE2	3.80
α13	246 (TYR)	OH	α2	38 (VAL)	CG1	3.86
α13	246 (TYR)	OH	α2	38 (VAL)	CG2	3.27
α13	250 (TYR)	CE2	α2	42 (GLU)	OE2	3.64
α13	250 (TYR)	OH	α2	42 (GLU)	OE2	3.33
α13	250 (TYR)	OH	α2	45 (ASN)	OD1	3.55
α13	253 (ARG)	NH1	α2	42 (GLU)	CD	3.84
α13	253 (ARG)	NH2	α2	42 (GLU)	CD	3.77
α13	253 (ARG)	CZ	α2	42 (GLU)	OE1	3.18
α13	253 (ARG)	NH1	α2	42 (GLU)	OE1	3.03
α13	253 (ARG)	NH2	α2	42 (GLU)	OE1	2.53
α13	253 (ARG)	NH1	α2	45 (ASN)	ND2	3.66

Supplementary Table 7. Bacterial strains used in this study.

Strains	Description	Reference
DH5α	Host for DNA cloning	
RN4220	Restriction-defective derivate of RN450	[19]
BL21 (DE3)	*E. coli* expression strain	Stratagene
Rosetta2	*E. coli* expression strain	Novagen
JP6774	RN4220 Δ*spa* SaPIbov1 *tst*::*tet*M	[20]
JP12666	RN4220 ϕNM1	This work
RN10616	RN4220 80α	[21]
JP8487	RN4220 ϕ11	This work
JP10280	JP6774 pJP1927	[2]
JP14649	JP6774 pJP1928	[2]
JP11742	JP6774 pJP2040	[2]
JP15291	JP6774 pCN51	[2]
JP18042	JP6774 pJP653	This work
JP19591	JP6774 ϕNM1	This work
JP19593	JP6774 ϕNM1$_{\Delta dut}$	This work
JP19592	JP6774 ϕ11	This work
JP19598	JP6774 ϕ11$_{\Delta dut}$	This work
JP19785	JP6774 80α	This work
JP19780	JP6774 80α$_{\Delta dut}$	This work
JP18043	JP6774 Stl^{Y234A}	This work
JP18044	JP18043 pJP653	This work
JP18045	JP18043 pJP1927	This work
JP18046	JP18043 pJP1928	This work
JP18047	JP18043 pJP2040	This work
JP18048	JP18043 pCN51	This work
JP18049	JP18043 ϕNM1	This work
JP19596	JP18043 ϕNM1$_{\Delta dut}$	This work
JP18050	JP18043 80α	This work
JP19783	JP18043 80α$_{\Delta dut}$	This work
JP18051	JP18043 ϕ11	This work
JP19601	JP18043 ϕ11$_{\Delta dut}$	This work
JP17679	JP6774 Stl^{HY-DA}	This work
JP18052	JP17679 pJP653	This work
JP18053	JP17679 pJP1927	This work
JP18054	JP17679 pJP1928	This work
JP18055	JP17679 pJP2040	This work
JP18056	JP17679 pCN51	This work
JP18057	JP17679 ϕNM1	This work

JP19597	JP17679 ϕNM1$_{\Delta dut}$	This work
JP18058	JP17679 80α	This work
JP19784	JP17679 80$\alpha_{\Delta dut}$	This work
JP18059	JP17679 ϕ11	This work
JP19602	JP17679 ϕ11$_{\Delta dut}$	This work
JP17043	JP6774 Stl^{Y112A}	This work
JP18060	JP17043 pJP653	This work
JP18061	JP17043 pJP1927	This work
JP18062	JP17043 pJP1928	This work
JP18063	JP17043 pJP2040	This work
JP18064	JP17043 pCN51	This work
JP18065	JP17043 ϕNM1	This work
JP19594	JP17043 ϕNM1$_{\Delta dut}$	This work
JP18066	JP17043 80α	This work
JP19781	JP17043 80$\alpha_{\Delta dut}$	This work
JP18067	JP17043 ϕ11	This work
JP19599	JP17043 ϕ11$_{\Delta dut}$	This work
JP17706	JP6774 Stl^{YYAA}	This work
JP18068	JP17706 pJP653	This work
JP18069	JP17706 pJP1927	This work
JP18070	JP17706 pJP1928	This work
JP18071	JP17706 pJP2040	This work
JP18072	JP17706 pCN51	This work
JP18073	JP17706 ϕNM1	This work
JP19595	JP17706 ϕNM1$_{\Delta dut}$	This work
JP18074	JP17706 80α	This work
JP19782	JP17706 80$\alpha_{\Delta dut}$	This work
JP18075	JP17706 ϕ11	This work
JP19600	JP17706 ϕ11$_{\Delta dut}$	This work
JP19771	RN4220 pJP2085	This work
JP19772	RN4220 pJP2086	This work
JP19773	RN4220 pJP2087	This work
JP19774	RN4220 pJP2172	This work
JP19775	RN4220 pJP2094	This work
JP19776	RN4220 pJP2090	This work
JP19777	RN4220 pJP2091	This work
JP19778	RN4220 pJP2173	This work
JP19779	RN4220 pJP2097	This work
JP14101	JP12666 pJP2085	This work
JP15719	JP12666 pJP2104	This work
JP14668	JP12666 pJP2100	This work
JP14661	JP12666 pJP2101	This work

JP13910	JP12666 pJP2105	This work
JP13907	JP12666 pJP2106	This work
JP17063	JP12666 pJP2176	This work
JP17064	JP12666 pJP2177	This work
JP17065	JP12666 pJP2178	This work
JP14103	RN10616 pJP2085	This work
JP15718	RN10616 pJP2104	This work
JP14666	RN10616 pJP2100	This work
JP14659	RN10616 pJP2101	This work
JP13911	RN10616 pJP2105	This work
JP13908	RN10616 pJP2106	This work
JP17057	RN10616 pJP2176	This work
JP17058	RN10616 pJP2177	This work
JP17059	RN10616 pJP2178	This work
JP14423	JP8487 pJP2085	This work
JP15717	JP8487 pJP2104	This work
JP14785	JP8487 pJP2100	This work
JP14786	JP8487 pJP2101	This work
JP14424	JP8487 pJP2105	This work
JP14425	JP8487 pJP2106	This work
JP17051	JP8487 pJP2176	This work
JP17052	JP8487 pJP2177	This work
JP17053	JP8487 pJP2178	This work

Supplementary Table 8. Plasmids used in this study.

Plasmid	Description	Reference
pPROEX HTa	Expression vector	Invitrogen
pLIC-SGC1	Expression vector	Invitrogen
pCN41	Expression vector	[22]
pCN51	Expression vector	[22]
pJP653	pCN51 3xflag 11 Dut^{WT}	[20]
pJP1927	pCN51 3xflag ΦNM1 Dut^{WT}	[2]
pJP1928	pCN51 3xflag ΦO11 Dut^{WT}	[2]
pJP2040	pCN51 3xflag ΦDI Dut^{WT}	[2]
pJP2085	pCN41 pInt-20^{WT}-19-18blaZ (SaPIbov1)	This work
pJP2086	pCN41 pInt-20^{R22A}-19-18blaZ (SaPIbov1)	This work
pJP2087	pCN41 pInt-20^{Q29A}-19-18blaZ (SaPIbov1)	This work
pJP2172	pCN41 pInt-20^{S44A}-19-18blaZ (SaPIbov1)	This work
pJP2094	pCN41 pInt-20^{N45A}-19-18blaZ (SaPIbov1)	This work
pJP2090	pCN41 pInt-20^{E47A}-19-18blaZ (SaPIbov1)	This work
pJP2091	pCN41 pInt-20^{N48A}-19-18blaZ (SaPIbov1)	This work
pJP2173	pCN41 pInt-20^{R51A}-19-18blaZ (SaPIbov1)	This work
pJP2104	pCN41 pInt-20^{Y112A}-19-18blaZ (SaPIbov1)	This work
pJP2100	pCN41 pInt-20^{Y113A}-19-18blaZ (SaPIbov1)	This work
pJP2101	pCN41 pInt-20^{Y116A}-19-18blaZ (SaPIbov1)	This work
pJP2105	pCN41 pInt-20^{Y106A}-19-18blaZ (SaPIbov1)	This work
pJP2106	pCN41 pInt-20$^{YY\text{-}AA}$-19-18blaZ (SaPIbov1)	This work
pJP2176	pCN41 pInt-20^{Y234A}-19-18blaZ (SaPIbov1)	This work
pJP2177	pCN41 pInt-20$^{HY\text{-}DA}$-19-18blaZ (SaPIbov1)	This work
pJP2178	pCN41 pInt-20^{GGGS}-19-18blaZ (SaPIbov1)	This work
pJP2097	pCN41 pInt-20^{R74A}-19-18blaZ (SaPIbov1)	This work
pJP666	pET28a Dut11	[23]
pJP753	pET28a Dut80a	[23]
pJP2046	pET28a DutDI	[2]
pJP2048	pET28a DutO11	[18]
pETNKI-Stl	pETNKI 1.10 Stl^{WT}	[15]
pAM1	pETNKI 1.10 Stl$^{N\text{-}ter}$	This work
pAM2	pETNKI 1.1 Stl$^{C\text{-}ter}$	This work
pAM3	pLIC-SGC1 Stl^{R22A}	This work
pAM4	pLIC-SGC1 Stl^{Q29A}	This work
pAM5	pLIC-SGC1 Stl^{S44A}	This work
pAM6	pLIC-SGC1 Stl^{N45A}	This work
pAM7	pLIC-SGC1 Stl^{E47A}	This work
pAM8	pLIC-SGC1 Stl^{N48A}	This work
pAM9	pLIC-SGC1 Stl^{R51A}	This work
pAM10	pLIC-SGC1 Stl^{H73C}	This work

pAM11	pLIC-SGC1 Stl^{R74A}	This work
pAM12	pLIC-SGC1 Stl^{Y112A}	This work
pAM13	pLIC-SGC1 Stl^{Y113A}	This work
pAM14	pLIC-SGC1 Stl^{YY-AA}	This work
pAM15	pLIC-SGC1 Stl^{Y116A}	This work
pAM16	pLIC-SGC1 Stl^{H188C}	This work
pAM17	pLIC-SGC1 Stl^{Y234A}	This work
pAM18	pLIC-SGC1 Stl^{HY-DA}	This work
pAM19	pLIC-SGC1 Stl^{GGGS}	This work
pAM20	pETNKI 1.10 $Stl^{Nter-H73C}$	This work

Supplementary Table 9. Oligonucleotide designs used in this study.

Plasmid	Oligonucleotides	Sequence
pJP653	orf32-phi80alpha-23mS	ACGCGTCGACATTATGGCAGGTCAAGTTGTCTATAAATATGAGGAGGAATAGGAAAATGGATTATAAAGATCACGATGGCGATTATAAAGATCACG
	orf32-phi80alpha-20m	TATAAAGATCACGATGGCGATTATAAAGATCACGATATCGATTATAAAGATGATGATGATAAAATGACTAACACATTACAAGTAAGGCTATTATCAGAA
	orf25-phi11-39cB	CGCGGATCCCTTTACACTCCGCTACTTCCG
pJP1928<br>pJP1927<br>pJP2040	dutNM1-1mS	ACGCGTCGACATTATGACGGGTCAAGTTGTCTATAAATATGAGGAGGCACAGGAAAATGGATTATAAAGATCACGATGGCGATTATAAAGATC
	dutNM1-2m	CACGATGGCGATTATAAAGATCACGATATCGATTATAAAGATGATGATGATAAAATGACTAACACATTAACAATTGATCAG
	dut-DI-2cB	CGCGGATCCTTACACGTATCCTTTTCCTGC<br>(These plasmids were constructed using the same primers with different templates.)
pJP2085	SaPIbov1-149cB	CGCGGATCCGATCAGTACCTAAATATGCG
	NY-24mK	CGGGGTACCCACTCGGTTATAACCTT
pJP2086*	SaPIbov1-orf20-34c	GTTAATTTCATGTATTTTGCAAGAGTTTTAATAATTG
	SaPIbov1-orf20-35m	CAATTATTAAAACTCTTGCAAAATACATGAAATTAAC
pJP2087*	SaPIbov1-orf20-36c	CACTCAATTTGCTTGCAGTTAATTTCATG
	SaPIbov1-orf20-37m	CATGAAATTAACTGCAAGCAAATTGAGTG
pJP2172*	SaPIbov1-orf20-80c	GCCGTTCTCGTGATTTGCAATGGTGTTTTGAC
	SaPIbov1-orf20-81m	GTCAAAACACCATTGCAAATCACGAGAACGGC
pJP2094*	SaPIbov1-orf-20-60c	GTTGCCGTTCTCGTGAGCTGAAATGGTGTTTTG
	SaPIbov1-orf-20-61m	CAAAACACCATTTCAGCTCACGAGAACGGCAAC
pJP2090*	SaPIbov1-orf20-40c	GTTTCTGTTGCCGTTCGCGTGATTTGAAATGG
	SaPIbov1-orf20-41m	CCATTTCAAATCACGCGAACGGCAACAGAAAC
pJP2091*	SaPIbov1-orf20-30c	GTTTCTGTTGCCGGCCTCGTGATTTG
	SaPIbov1-orf20-31m	CAAATCACGAGGCCGGCAACAGAAAC
pJP2173*	SaPIbov1-orf20-78c	CATTTACTCCAATGTTTGCGTTGCCGTTCTCGTG
	SaPIbov1-orf20-79m	CACGAGAACGGCAACGCAAACATTGGAGTAAATG

pJP2104*	SaPIbov1-orf20-50c<br>SaPIbov1-orf20-51m	CGTATGATGAATAAGCAATGTCGCCGTC<br>GACGGCGACATTGCTTATTCATCATACG
pJP2100*	SaPIbov1-orf20-52c<br>SaPIbov1-orf20-53m	CATATAAATCGTATGATGAAGCATAAATGTCGCCGTC<br>GACGGCGACATTTATGCTTCATCATACGATTTATATG
pJP2101*	SaPIbov1-orf20-54c<br>SaPIbov1-orf20-55m	GTTTCATCATATAAATCGGCTGATGAATAATAAATG<br>CATTTATTATTCATCAGCCGATTTATATGATGAAAC
pJP2105*	SaPIbov1-orf20-16c<br>SaPIbov1-orf20-15m	ATAAATGTCGCCGTCATTTGCATAGGCTTTATTAACATAA<br>TTATGTTAATAAAGCCTATGCAAATGACGGCGACATTTAT
pJP2106*	SaPIbov1-orf20-18c<br>SaPIbov1-orf20-17m	TATAAATCGTATGATGATGCTGCAATGTCGCCGTCATTAT<br>ATAATGACGGCGACATTGCAGCATCATCATACGATTTATA
pJP2176*	SaPIbov1-orf20-90c<br>SaPIbov1-orf20-91m	CCTTTAATAGCATCAGCATGATGGTTAGGAACC<br>GGTTCCTAACCATCATGCTGATGCTATTAAAGG
pJP2177*	SaPIbov1-orf20-97c<br>SaPIbov1-orf20-96m	CCTTTAATAGCATCAGCATGATCGTTAGGAACC<br>GGTTCCTAACGATCATGCTGATGCTATTAAAGG
pJP2178*	SaPIbov1-orf20-106c<br>SaPIbov1-orf20-107m	CATTGGGCTACCTCCTCCAGGAACC<br>GGTTCCTGGAGGAGGTAGCCCAATG
pJP2097*	SaPIbov1-orf20-42c<br>SaPIbov1-orf20-43m	CTTTAAACTCATCTGATATCGCGTGTAGAATATAGCTGGG<br>CCCAGCTATATTCTACACGCGATATCAGATGAGTTTAAAG
pAM1	Stl_Nter_STOP_Fw<br>Stl_Nter_STOP_Rv	CACTGATACATAAAAATCAATAATAAATTACG<br>GAAAGGATTTGTTTGTACAAC
pAM2	FwStl1-8_K175-N267<br>RvStlpETNKI1-8	CAGGGACCCGGTAAAAAAAGAGAAGTAACAATAGAAG<br>CGAGGAGAAGCCCGGTTAATTAGTGTCTTTTTCAAGTATG
pAM3 to pAM15	Stl_OFA_Fw<br>Stl_OFA_Rv	TACTTCCAATCCATGATGGAAGGAGCTGGTCAAATG<br>TATCCACCTTTACTGTCATTAATTAGTGTCTTTTTCAAGTATG<br>(These plasmids were constructed using the same primers with different templates.)
pAM16	Stl_H188C_Fw<br>Stl_H188C_Rv	TGGTGAATTTTGCGAAAAATATTTAAAACTATTATTC<br>ATTTCTTCTATTGTTACTTCTC

pAM17 to pAM19	SaPIbov1-orf20-102m<br>SaPIbov1-orf20-103c	TACTTCCAATCCATGATGGAAGGAGCTGGTCAAATGGC<br>TATCCACCTTTACTGTTAATTAGTGTCTTTTTCAAGTATG<br>(These plasmids were constructed using the same primers with different templates.)
pAM20	Stl_NterH73C_Fw<br>Stl_NterH73C_Rv	TATATTCTATGCAGAATATCAGATGAGTTTAAAGAAAAAG<br>CTGGGTATACCTAAACCTTTAC
**Probe**	**Oligonucleotides**	**Sequence**
**SaPIbov1**	SaPIbov1-112mE<br>SaPIbov1-113cB	CCGGAATTCAATTGCTGAGGCAAAACTTC<br>CGCGGATCCTAATTCTCCACGTCTAAAGC
**SaPIbov1**	Stl_DNA_BR1<br>Stl_DNA_BR2	AAACATATTCTCACCTCCTCG<br>TAAATCCTGTCCTTTCACTCAA

Supplementary References

1 Vertessy, B. G. & Toth, J. Keeping uracil out of DNA: physiological role, structure and catalytic mechanism of dUTPases. *Acc Chem Res* **42**, 97-106, doi:10.1021/ar800114w (2009).

2 Donderis, J. *et al.* Convergent evolution involving dimeric and trimeric dUTPases in pathogenicity island mobilization. *PLoS Pathog* **13**, e1006581, doi:10.1371/journal.ppat.1006581 (2017).

3 Moroz, O. V. *et al.* Dimeric dUTPases, HisE, and MazG belong to a new superfamily of all-alpha NTP pyrophosphohydrolases with potential "house-cleaning" functions. *J Mol Biol* **347**, 243-255, doi:10.1016/j.jmb.2005.01.030 (2005).

4 Barabas, O. *et al.* Catalytic mechanism of alpha-phosphate attack in dUTPase is revealed by X-ray crystallographic snapshots of distinct intermediates, 31P-NMR spectroscopy and reaction path modelling. *Nucleic Acids Res* **41**, 10542-10555, doi:10.1093/nar/gkt756 (2013).

5 Hemsworth, G. R., Gonzalez-Pacanowska, D. & Wilson, K. S. On the catalytic mechanism of dimeric dUTPases. *Biochem J* **456**, 81-88, doi:10.1042/BJ20130796 (2013).

6 Leveles, I. *et al.* Structure and enzymatic mechanism of a moonlighting dUTPase. *Acta Crystallogr D Biol Crystallogr* **69**, 2298-2308, doi:10.1107/S0907444913021136 (2013).

7 Poornam, G. P., Matsumoto, A., Ishida, H. & Hayward, S. A method for the analysis of domain movements in large biomolecular complexes. *Proteins* **76**, 201-212, doi:10.1002/prot.22339 (2009).

8 Pecsi, I. *et al.* Nucleotide pyrophosphatase employs a P-loop-like motif to enhance catalytic power and NDP/NTP discrimination. *Proc Natl Acad Sci U S A* **108**, 14437-14442, doi:10.1073/pnas.1013872108 (2011).

9 Holm, L. & Laakso, L. M. Dali server update. *Nucleic Acids Res* **44**, W351-355, doi:10.1093/nar/gkw357 (2016).

10 McGeehan, J. E. *et al.* Structural analysis of the genetic switch that regulates the expression of restriction-modification genes. *Nucleic Acids Res* **36**, 4778-4787, doi:10.1093/nar/gkn448 (2008).

11 Papp-Kadar, V., Szabo, J. E., Nyiri, K. & Vertessy, B. G. In Vitro Analysis of Predicted DNA-Binding Sites for the Stl Repressor of the Staphylococcus aureus SaPIBov1 Pathogenicity Island. *PLoS One* **11**, e0158793, doi:10.1371/journal.pone.0158793 (2016).

12 Ball, N. J., McGeehan, J. E., Streeter, S. D., Thresh, S. J. & Kneale, G. G. The structural basis of differential DNA sequence recognition by restriction-modification controller proteins. *Nucleic Acids Res* **40**, 10532-10542, doi:10.1093/nar/gks718 (2012).

13 Dodd, I. B., Shearwin, K. E. & Egan, J. B. Revisited gene regulation in bacteriophage lambda. *Curr Opin Genet Dev* **15**, 145-152, doi:10.1016/j.gde.2005.02.001 (2005).

14 Lewis, R. J., Brannigan, J. A., Offen, W. A., Smith, I. & Wilkinson, A. J. An evolutionary link between sporulation and prophage induction in the structure of a repressor:anti-repressor complex. *J Mol Biol* **283**, 907-912, doi:10.1006/jmbi.1998.2163 (1998).

15 Maiques, E. *et al.* Another look at the mechanism involving trimeric dUTPases in Staphylococcus aureus pathogenicity island induction involves novel players in the party. *Nucleic Acids Res* **44**, 5457-5469, doi:10.1093/nar/gkw317 (2016).

16 Szabo, J. E. *et al.* Highly potent dUTPase inhibition by a bacterial repressor protein reveals a novel mechanism for gene expression control. *Nucleic Acids Res* **42**, 11912-11920, doi:10.1093/nar/gku882 (2014).

17 Sagendorf, J. M., Berman, H. M. & Rohs, R. DNAproDB: an interactive tool for structural analysis of DNA-protein complexes. *Nucleic Acids Res* **45**, W89-W97, doi:10.1093/nar/gkx272 (2017).

18 Bowring, J. *et al.* Pirating conserved phage mechanisms promotes promiscuous staphylococcal pathogenicity island transfer. *Elife* **6**, doi:10.7554/eLife.26487 (2017).

19 Krissinel, E. & Henrick, K. Inference of macromolecular assemblies from crystalline state. *J Mol Biol* **372**, 774-797, doi:10.1016/j.jmb.2007.05.022 (2007).

20 Tormo-Mas, M. A. *et al.* Moonlighting bacteriophage proteins derepress staphylococcal pathogenicity islands. *Nature* **465**, 779-782, doi:10.1038/nature09065 (2010).

21 Ubeda, C. *et al.* Specificity of staphylococcal phage and SaPI DNA packaging as revealed by integrase and terminase mutations. *Mol Microbiol* **72**, 98-108 (2009).

22 Charpentier, E. *et al.* Novel cassette-based shuttle vector system for gram-positive bacteria. *Appl Environ Microbiol* **70**, 6076-6085, doi:10.1128/AEM.70.10.6076-6085.2004 (2004).

23 Tormo-Mas, M. A. *et al.* Phage dUTPases control transfer of virulence genes by a proto-oncogenic G protein-like mechanism. *Mol Cell* **49**, 947-958, doi:10.1016/j.molcel.2012.12.013 (2013).
