# Supplementary material for: The structure of a polygamous repressor reveals how phage-inducible chromosomal islands spread in nature
Source: Nat Commun. 2019 Aug 15;10:3676. doi: 10.1038/s41467-019-11504-2 (PMC6695447; doi:10.1038/s41467-019-11504-2)
Supplement: Supplementary file 3 — Reporting Summary [file 41467_2019_11504_MOESM3_ESM.pdf]

nature research

Corresponding author(s): Alberto Marina and José R Penadés

Reporting Summary

Nature Research wishes to improve the reproducibility of the work that we publish. This form provides structure for consistency and transparency in reporting. For further information on Nature Research policies, see Authors & Referees and the Editorial Policy Checklist.

Statistical parameters

When statistical analyses are reported, confirm that the following items are present in the relevant location (e.g. figure legend, table legend, main text, or Methods section).

n/a	Confirmed	
☐	☒	The exact sample size (n) for each experimental group/condition, given as a discrete number and unit of measurement
☐	☒	An indication of whether measurements were taken from distinct samples or whether the same sample was measured repeatedly
☐	☒	The statistical test(s) used AND whether they are one- or two-sided<br>*Only common tests should be described solely by name; describe more complex techniques in the Methods section.*
☐	☒	A description of all covariates tested
☐	☒	A description of any assumptions or corrections, such as tests of normality and adjustment for multiple comparisons
☐	☒	A full description of the statistics including central tendency (e.g. means) or other basic estimates (e.g. regression coefficient) AND variation (e.g. standard deviation) or associated estimates of uncertainty (e.g. confidence intervals)
☐	☒	For null hypothesis testing, the test statistic (e.g. F, t, r) with confidence intervals, effect sizes, degrees of freedom and P value noted<br>*Give P values as exact values whenever suitable.*
☒	☐	For Bayesian analysis, information on the choice of priors and Markov chain Monte Carlo settings
☒	☐	For hierarchical and complex designs, identification of the appropriate level for tests and full reporting of outcomes
☒	☐	Estimates of effect sizes (e.g. Cohen's d, Pearson's r), indicating how they were calculated
☐	☒	Clearly defined error bars<br>*State explicitly what error bars represent (e.g. SD, SE, CI)*

Our web collection on statistics for biologists may be useful.

Software and code

Policy information about availability of computer code

Data collection	n/a
Data analysis	Size exclussion chromatogram figures, Fluorescence intensity analysis in Thermal Shift Assay , and Statistical Analyses were carried out with the scientific software GraphPad Prism. Open source software (https://www.graphpad.com/scientific-software/prism/)<br><br>Crystallographic processing was carried out with programs from CCP4 and Phenix, free and open source suites (http://www.ccp4.ac.uk/ ; https://www.phenix-online.org/).<br><br>Size Exclusion Chromatography with Multi-Angle Light Scattering (SEC-MALS) data acquistion and analysis was carried out with Astra 7.1.2 software (https://www.wyatt.com/products/software/astra.html) from Wyatt<br><br>Biolayer interferometry (BLI) data acquistion and analysis was carried out with BLItz Pro 1.2 software (https://www.blitzmenow.com/blitz_pro.html) from ForteBio.<br><br>MicroScale Thermophoresis (MST) data acquistion and analysis was carried out with M.O. Affinity Analysis software (https://nanotempertech.com/monolith-mo-control-software/) from NanoTermper Technologies<br><br>Protein asseblies and interactions analysis were carried out using the indicated PDBs and the webservers PDBePISA (http://www.ebi.ac.uk/pdbe/pisa/), PDBsum (http://www.ebi.ac.uk/thornton-srv/databases/cgi-bin/pdbsum/GetPage.pl?pdbcode=index.html)

April 2018

and CONTACT (http://www.ccp4.ac.uk/html/contact.html) software from CCP4suite (http://www.ccp4.ac.uk/).

Structural models were minimized with Chimera (http://www.cgl.ucsf.edu/chimera/) or Yasara (http://www.yasara.org) softwares.

DNA-protein contacs were anaylyzed with DNAproDB software (http://dnaprodb.usc.edu).

Figures for three-dimensional structures were generated with Pymol (https://pymol.org/2/) or Chimera (http://www.cgl.ucsf.edu/chimera/) softwares.

All this information has been included in the manuscript.

For manuscripts utilizing custom algorithms or software that are central to the research but not yet described in published literature, software must be made available to editors/reviewers upon request. We strongly encourage code deposition in a community repository (e.g. GitHub). See the Nature Research guidelines for submitting code & software for further information.

Data

Policy information about availability of data

All manuscripts must include a data availability statement. This statement should provide the following information, where applicable:

- Accession codes, unique identifiers, or web links for publicly available datasets
- A list of figures that have associated raw data
- A description of any restrictions on data availability

Protein structures are deposited in the public database "RCSB-Protein Data Bank" (https://www.rcsb.org/) with the accession codes: 6H48 ; 6H49; 6H4B ; 6H4C

Field-specific reporting

Please select the best fit for your research. If you are not sure, read the appropriate sections before making your selection.

☒ Life sciences ☐ Behavioural & social sciences ☐ Ecological, evolutionary & environmental sciences

For a reference copy of the document with all sections, see nature.com/authors/policies/ReportingSummary-flat.pdf

Life sciences study design

All studies must disclose on these points even when the disclosure is negative.

Sample size	Three independent experiment were carried out in the beta-lactamase assay.
Data exclusions	No data was excluded from the analyses.
Replication	Three replicates of the phage spot tests were performed. Error bars in beta-lactamase assays are calculated from three independent experiments.<br><br>At least three replicates were carried out for each sample in Native-PAGE gels and EMSA assays.<br><br>Binding affinities were measured by BLI or MST two times for each sample. No discrepancies superior of 10 % were observed between samples.
Randomization	Not relevant for our study. Samples not allocated in sample groups. Independent bacterial cultures for each bacterial strain were cultured.
Blinding	n/a

Reporting for specific materials, systems and methods

Materials & experimental systems

n/a	Involved in the study	
☒	☐	Unique biological materials
☐	☒	Antibodies
☒	☐	Eukaryotic cell lines
☒	☐	Palaeontology
☒	☐	Animals and other organisms
☒	☐	Human research participants

Methods

n/a	Involved in the study	
☒	☐	ChIP-seq
☒	☐	Flow cytometry
☒	☐	MRI-based neuroimaging

Antibodies

Antibodies used	Monoclonal ANTI-FLAG 685 M2-Peroxidase (HRP) antibody from Sigma-Aldrich was used in western blot assays.
Validation	This antobody has been extensively used and characterised in our previous studies.
